# Translation and validation of the Arabic version of the osteoarthritis quality of life questionnaire (OAQoL) in Saudi patients with osteoarthritis

**DOI:** 10.1186/s12955-021-01741-9

**Published:** 2021-03-17

**Authors:** Mansour AlAjmi, Sameer Al-Ghamdi

**Affiliations:** Department of Family and Community Medicine, College of Medicine, Prince Sattam bin Abdulaziz University, Al Kharj 11942, Saudi Arabia

**Keywords:** Osteoarthritis, OAQoL, Osteoarthritis quality of life scale, Health related quality of life

## Abstract

**Background:**

Osteoarthritis (OA) is a debilitating multifactorial degenerative rheumatic disease affecting millions of people around the globe. The osteoarthritis quality of life scale (OAQoL), originally produced in the English language, is an important tool used to assess the overall impact of OA and its treatment on the patient’s quality of life.

**Purpose:**

The purpose of the study was to translate and validate the OAQoL in the Arabic language in order to use it on the Saudi population.

**Methodology:**

A bilingual panel comprising four healthcare professionals and one external certified medical translator translated the English version of the OAQoL to the Arabic language. A back translation was subsequently performed by two English-speaking translators and any differences were resolved by conferring with the original panel. The qualitative research was performed through cognitive debriefing interviews (CDIs) with 59 native Arabic patients who had clinically and radiologically confirmed osteoarthritis of any joint. The internal consistency of the 22 items was derived by leveraging the Cronbach’s Alpha coefficient.

**Results:**

59 participants were included in the study, and more than half (52.5%) of them were men. The response rate was 100% and the mean time taken to answer the questionnaire was 10.5 min. The average Intraclass Correlation Coefficient (ICC) and Cronbach’s Alpha were determined to be 0.93 each, indicating that all the items in the OAQoL were significantly interrelated.

**Conclusion:**

The translated Arabic version of the OAQoL questionnaire used in this study is a reliable and consistent tool that showed good comprehensibility and internal consistency.

## Background

Osteoarthritis (OA) is a debilitating multifactorial degenerative rheumatic disease, and one of the leading causes of functional limitations and morbidity worldwide [1]. Progressive degeneration of joints results in physical disability affecting 250 million individuals around the globe [2]. It has been estimated that 9.6% of men and 18% of women over 60 years of age suffer from symptomatic OA [3]. It has been reported that people with OA of the knees contribute to the 3.8% prevalence of symptomatic OA worldwide [4]. The prevalence of OA in the Arab world is significantly high; contributing to 53.3% of men and 60.9% of women between 30 and 93 years of age suffering from radiographically confirmed symptomatic knee OA in the Middle East [5]. The prevalence of OA in Saudi Arabia is 30.8% and 60.6% in people aged 46–55 years and 66–75 years [6].

Pain and disability in patients with OA limit their daily activities and significantly affect their quality of life (QoL) and socioeconomic status [7]. The ultimate goal of medical practice is to maintain health related quality of life. In order to assess the quality of life the practitioner needs to ask the patients questions about the physical, mental, and psychosocial aspects of the disease [8]. Quality of life instruments or questionnaires are developed to reduce bias among practitioners. Therefore, a variety of assessment questionnaires or tools is used to determine pain, disability, and QoL among patients with OA [5]. For example, the osteoarthritis quality of life scale (OAQoL), the 36-item short form survey (SF-36), the EuroQoL instrument (EQ-5D), the knee injury and osteoarthritis outcome score (KOOS), the World Health Organization quality of life (WHOQOL-100), the Japanese knee osteoarthritis measure (JKOM), and the arthritis impact measurement scales (AIMS) are used to assess the quality of life of patients suffering from OA [3, 9–14]. The OAQoL is one of the important tools used to assess the overall impact of OA and its treatment on patients’ quality of life [3]. The OAQoL was developed in English in the United Kingdom (UK), and consists of 22 items to measure the QoL specific to OA [15]. The OAQoL assessment tool helps assess the needs of patients with OA in terms of cognition, esteem, fulfilment, safety, security, belongingness, love, and physical strength [15]. It has good psychometric qualities and is validated for the assessment of OA involving the upper and lower limbs, as well as their combination [3]. It is a common, clear, and easy assessment tool to use for outdoor patients [16]. Due to its excellent internal consistency and construct validity the English version of the OAQoL has been translated and validated in several languages in order to assess the need-based QoL among patients suffering from OA in various regions of the world [3].

Cross-cultural adaptation of an assessment tool may face problems of linguistic and conceptual equivalence. Some phrases may be recognized differently in different languages. Cross-cultural translation and validation of health-related assessment tools therefore involves translators and bilingual authors [17]. Some studies have used dual panel methodology during the cross-cultural translation and validation of the OAQoL where two panels are conducted—a bilingual panel to translate the questionnaire and a lay panel to assess the comprehension of the language [16, 18]. Having a bilingual panel expert is recommended by the World Health Organization (WHO) in order to identify and resolve any inadequate concepts, expressions, or discrepancies found between the two versions of the questionnaire or tool [19]. The purpose of using a bilingual panel is to produce a questionnaire in another language where the questions are understood in the same way in each language. However, consistency and reproducibility is assessed later to validate the new version.

The OAQoL questionnaire has been translated and validated in Portuguese, German, Hungarian, Spanish, French, Italian, and Turkish [3, 16, 20]. Despite high prevalence of OA in Arabic countries an Arabic version of the OAQoL questionnaire was not available to validate this useful tool to assess the quality of Arabic patients suffering from OA. The English version of the OAQoL questionnaire was therefore translated to Arabic by a bilingual panel in order to determine its validity in the Arabic population suffering from OA. This study is a useful addition to literature in terms of a simple and true patient-based conceptual tool for clinicians working in Arabic areas where they can assess OA patients with ease.

## Methodology

### The osteoarthritis quality of life questionnaire translation process

This study sought to translate the Osteoarthritis Quality of Life Questionnaire (OAQoL) into the Arabic language for its subsequent validation for use on Arabic patients. A bilingual panel consisting of one orthopedic surgeon, one physiotherapist, one behavioral psychologist, one general practitioner, and an external certified medical translator was assembled to translate the OAQoL into Arabic. The members of the panel were chosen to represent a diverse spectrum of healthcare professionals who manage OA in their professional capacity. The external medical translator was included in the panel so that any differences delineated by the other members could be resolved. All members of the panel were native Arabs and spoke and read English fluently. Back translation of the Arabic OAQoL was performed as an additional measure. The Arabic version of the OAQoL was translated by two English speaking translators who were blind to the aims and objectives of this study. Differences between the Arabic OAQoL and the back-translated English OAQoL were resolved after the original panel conferred with the two translators.

### Patient enrollment

Convenience sampling was conducted at the general medical and orthopedic outpatient clinics of the Military Industries Corporation Hospital in Al Kharj, Saudi Arabia. 59 patients were recruited for this study. A review of the literature reveals that the validation of the OAQoL has been performed in studies that recruited between 17 and 53 patients. Specific inclusion and exclusion criteria were designed and applied to the participants. The inclusion criteria were: male and female native Arabs, literate and fluent in Arabic, capable of providing informed consent, and having formally clinically and radiologically diagnosed osteoarthritis of any joint. The exclusion criteria were: visual disturbances which precluded filling in the questionnaire (e.g. severe retinopathy or cataracts), lacking mental capacity as per the Fraser Gillick guidelines, osteoarthritis secondary to rheumatological conditions such as rheumatoid arthritis or psoriatic arthropathy, other significant co-morbidities that could affect the quality of life (e.g. advanced chronic obstructive pulmonary disease, cancer, and congestive cardiac failure), and patients who were acutely unwell to a point that warranted admission to the inpatient setting. Recruitment commenced on 14 January 2019 and the study concluded on 14 January 2020.

### Patient interviews

Qualitative research was performed by interviewing 59 native Arabic patients with clinically and radiologically confirmed osteoarthritis of any joint (e.g., hip, knee, shoulder, proximal interphalangeal joints).

The questionnaire comprises 22 questions which contend with the patient’s social activities, level of enjoyment, social inclusion, feelings of embarrassment and disappointment, and other aspects. The questionnaire has been appended to this paper as a supplementary file.

The study was approved by the Institutional Review Board (IRB) of the Prince Sattam Bin Abdulaziz University. Informed consent was obtained from patients recruited for the study, and they were provided the questionnaires to answer in the outpatient setting after their consultation at the general medical or orthopedic outpatient clinics.

Cognitive debriefing interviews (CDIs) were conducted with all 59 patients to assess their comprehension and the relevance of the translated OAQoL. During these interviews patients were asked about the following aspects: the comprehension of each of the 22 questions of the questionnaire, the type of information they needed to recall to answer the questions, and the decision processes undertaken by participants to answer the questions accurately. Participants were also requested to provide general comments on the questionnaire (e.g., the questionnaire being too long). The CDIs were conducted with the ‘think aloud’ method which was deliberately chosen in an effort to mitigate interviewer bias. All 59 participants were instructed to think aloud as they answered the 22 questions, and the data were transcribed manually by the interviewers. The ‘think aloud’ method was chosen to determine whether the meaning of each of the 22 questions as intended by the OAQOL was consistent with the participants’ interpretation of the item.

### Ethical considerations

The study was approved by the Institutional Review Board (IRB) of the Prince Sattam Bin Abdulaziz University. Informed consent was obtained from patients recruited for the study, and they were provided the questionnaires to answer in the outpatient setting after their consultation at the general medical or orthopedic outpatient clinics.

### Data collection and analysis

All data from the questionnaire were entered into Microsoft Excel 2013 and subsequently analyzed with the SPSS (Statistical Package Social Science) software version 20. Demographic characteristics such as age and gender were obtained and described. The patients’ personal identifiers were deidentified to ensure that their privacy and confidentiality were not compromised. The frequency and percentages of the OAQoL responses were measured. The mean and standard deviation were calculated for age and scale responses. The intra-item correlation was computed. The internal consistency of the 22 items was derived by leveraging the Cronbach’s Alpha coefficient. The item total correlation, the total Alpha value, and the Alpha value of each item were estimated. Scores exceeding 0.8 are desirable, scores exceeding 0.90 are excellent.

All data from the interviews were manually transcribed by three interviewers and subjected to a grounded theory analysis. The grounded theory analysis is a validated method which enables researchers to delineate concepts and generate theory from qualitative data. The data from the interviews were coded and analyzed to generate themes.

## Results

52.5% of the 59 participants in this study were men and 47.5% were women. Most of the participants were middle-aged and older adults—66.1% of them were between the age of 41 and 60. The specific demographic details of patients are delineated in Table 1. A full review of all the responses of the OAQoL can be found in Table 2.

**Table 1:** Participant demographic characteristics

Study variables	Frequency	Percent (%)
Age (in years)		
Mean +/− SD	48.4 +/− 11.3	
≤ 20 years	1	1.7
21–30 years	3	5.1
31–40 years	10	16.9
41–50 years	19	32.2
51–60 years	20	33.9
> 60 years	6	10.2
Gender		
Female	28	47.5
Male	31	52.5
Total	59	100

**Table 2:** OAQoL response frequencies

Questions		Frequency	Percentage (%)
I’m unable to join in activities with my friends or family	No	34	57.6
	Yes	25	42.4
I get embarrassed using stairs in public	No	27	46.6
	Yes	31	53.4
I feel like I am missing out on life	No	36	61.0
	Yes	23	39.0
I can’t plan things too far in advance	No	41	69.5
	Yes	18	30.5
I feel as though I’m trapped in my house	No	46	78.0
	Yes	13	22.0
My arthritis limits the places I can go	No	7	11.9
	Yes	52	88.1
I can’t do things on the spur of the moment	No	21	35.6
	Yes	38	64.4
It interferes with everything I do	No	28	48.3
	Yes	31	52.5
Walking for pleasure is out of the question	No	31	52.5
	Yes	28	47.5
I can’t enjoy myself when I go out	No	33	55.9
	Yes	26	44.1
I feel useless	No	50	84.7
	Yes	9	15.3
I feel I can’t join in with social activities	No	35	59.3
	Yes	24	40.7
My arthritis controls my life	No	33	55.9
	Yes	26	44.1
I feel dependant on others	No	40	67.8
	Yes	19	32.2
I worry about being a nuisance to other people	No	26	44.1
	Yes	33	55.9
My life revolves around my arthritis	No	39	66.1
	Yes	20	33.9
I can’t be as independent as I want	No	23	39.0
	Yes	36	61.0
I feel very isolated	No	49	83.1
	Yes	10	16.9
I can’t live life to the full	No	22	37.3
	Yes	37	62.7
I have to limit what I do each day	No	14	23.7
	Yes	45	76.3
I feel slowed down	No	36	61.0
	Yes	23	39.0
I can’t go to the places I want to go	No	37	62.7
	Yes	22	37.3

Three interviewers conducted the CDIs for all 59 participants—the mean duration of the interviews was 11.3 min. Two main themes arose from the grounded theory analysis performed on the qualitative data transcribed by the interviewers. Firstly, 17 of the 59 participants opined that the questionnaire was too long. 8 participants suggested that questions could be combined to generate a more succinct questionnaire. Secondly, most of the questions were understood as intended. The feedback collated from participants showed that only 1 out of 22 questionnaires was frequently misinterpreted. The question which was frequently misinterpreted was ‘I feel slowed down’. 13 of the 59 participants interpreted this as slowing down of the pace of life, rather than slowing of movement or ambulation. After the 13th participant articulated their opinions of this question, it was adapted in consultation with the original bilingual panel convened for the translation of the OAQOL as well as both the English translators. This issue did not recur after the adaptation of this question for the subsequent interviews. Cronbach’s Alpha was used to measure the internal consistency of the questionnaire. Internal consistency refers to the degree to which all 22 items in the OAQoL measure the same concept/construct, and is therefore linked to the interrelatedness of the items. Cronbach’s Alpha was determined to be 0.93, indicating that all 22 items in the OAQoL were significantly interrelated. The derivation of Cronbach’s Alpha for all items can be found in Table 3.

**Table 3:** Reliability and validity analysis of OAQoL items

Questions	Scale mean	Scale variance	Corrected correlation	Cronbach's alpha
1. I’m unable to join in activities with my friends or family	9.56	39.04	0.642	0.926
2. I get embarrassed using stairs in public	9.46	39.08	0.629	0.927
3. I feel like I am missing out on life	9.59	38.93	0.670	0.926
4. I can’t plan things too far in advance	9.68	39.32	0.644	0.926
5. I feel as though I’m trapped in my house	9.76	40.04	0.581	0.927
6. My arthritis limits the places I can go	9.10	41.98	0.283	0.931
7. I can’t do things on the spur of the moment	9.34	40.12	0.480	0.929
8. It interferes with everything I do	9.46	38.18	0.780	0.924
9. Walking for pleasure is out of the question	9.51	38.42	0.739	0.924
10. I can’t enjoy myself when I go out	9.54	38.52	0.726	0.925
11. I feel useless	9.83	40.41	0.595	0.927
12. I feel I can’t join in with social activities	9.58	38.59	0.724	0.925
13. My arthritis controls my life	9.54	38.25	0.773	0.924
14. I feel dependent on others	9.66	38.33	0.812	0.923
15. I worry about being a nuisance to other People	9.42	41.62	0.219	0.934
16. My life revolves around my arthritis	9.64	40.30	0.456	0.930
17. I can’t be as independent as I want	9.37	40.75	0.366	0.931
18. I feel very isolated	9.81	39.77	0.707	0.926
19. I can’t live life to the full	9.36	40.88	0.347	0.932
20. I have to limit what I do each day	9.22	41.79	0.237	0.933
21. I feel slowed down	9.59	38.24	0.790	0.924
22. I can’t go to the places I want to go	9.61	38.03	0.834	0.923
Overall (for 22 items)	9.98	43.29	0.93	0.93

## Discussion

This study describes the translation and validation of the OAQoL with excellent internal consistency and reliability in the Arabic language in order to assess the quality of life of patients with OA living in Saudi Arabia. The bilingual panel and English-speaking translators produced a well regulated questionnaire which was well understood by the patients during cognitive debriefing interviews (CDIs). However, during the interview the participants complained that the questionnaire was long. Cronbach’s Alpha produced acceptable internal consistency of the Arabic form of the questionnaire. During the production of the Arabic version the conceptual equivalence was adapted instead of linguistic equality so that questions included in the questionnaire could be delineated in the Arabic language in the same way as in the English language.

Gomes et al. [16] translated and validated the OAQoL questionnaire in Portuguese with the Cronbach’s Alpha score of 0.87 for internal consistency. They also tested and retested the reliability of the questionnaire through CDIs and the Spearman’s rank correlation coefficient. In this study the Cronbach’s Alpha score was 0.93 which indicates excellent internal consistency of the Arabic version of the OAQoL questionnaire. It demonstrates that this study produced a more consistent and reliable Arabic version of the OAQoL compared to the Portuguese version. Differences in the Alpha score can be statistically attributed to interitem covariances or interrelatedness of the items [21]. Wilburn et al. [3] translated, debriefed, and validated the OAQoL questionnaire in German, Hungarian, Italian, Spanish, and Turkish with the Cronbach’s Alpha score ranging between 0.94 and 0.97 and the test–retest reliability (Spearman’s rank correlation) being 0.87 to 0.98. Similarly, Couraud et al. [20] translated, debriefed, and validated the OAQoL questionnaire in French with the Cronbach’s Alpha at 0.91 and the test–retest reliability at 0.93. This study has therefore produced an excellent, reliable, and valid Arabian version of the OAQoL questionnaire.

### Strengths and limitations

This study is the first effort to reproduce a reliable and consistent tool for easy and prompt clinical assessment of OA patients in Saudi Arabia. Involvement of the bilingual panel and English speakers in the translation of the questionnaire items to avoid inadequate expressions and discrepancies indicates the methodological strength of this study. Limitations of the study include the limited sample size, single-centred study, and absence of cross analysis of age and gender as these factors affect the quality of life of the patients with OA. In addition, test–retest reliability was not assessed in this study. Moreover, some participants complained that the questionnaire was time-consuming. In this regard a more succinct questionnaire can be produced through further research. Therefore future studies can be conducted with larger samples at multiple centers for further evaluation of the Arabic version in terms of its reliability and internal consistency.

## Conclusion

The translated Arabic version of the OAQoL questionnaire used in this study is a reliable and consistent tool that showed good comprehensibility and internal consistency. However, the study focused on the Cronbach’s alpha for the new Arabic version of the OAQoL which offered a reliable measure of quality of life of patients with OA. In this context other psychometric properties need to be evaluated to strengthen the new version of the questionnaire. The new version can be considered to assess patients with OA in a routine clinical setup as well as to conduct OA-related research among the Saudi population. This is a pilot study and with the help of more multicenter robust studies clinicians working in Arabic countries like Saudi Arabia may be equipped with an advanced tool to determine the quality of life of patients suffering from OA as well as to investigate the impact of interventions.

## Data Availability

Data are available upon request from the author.
